# Hybrid Radiobioconjugated Superparamagnetic Iron Oxide-Based Nanoparticles for Multimodal Cancer Therapy

**DOI:** 10.3390/pharmaceutics13111843

**Published:** 2021-11-02

**Authors:** Michał Żuk, Weronika Gawęda, Agnieszka Majkowska-Pilip, Magdalena Osial, Marcin Wolski, Aleksander Bilewicz, Paweł Krysiński

**Affiliations:** 1 Faculty of Chemistry, University of Warsaw, Pasteura 1 Str., 02-093 Warsaw, Poland; mt_zuk@chem.uw.edu.pl (M.Ż.); mosial@chem.uw.edu.pl (M.O.); 2 Institute of Nuclear Chemistry and Technology, Dorodna 16 Str., 03-195 Warsaw, Poland; w.maliszewska@ichtj.waw.pl (W.G.); a.majkowska@ichtj.waw.pl (A.M.-P.); 3 Centre for Radiotherapy Amethyst, Lubańska 11-12, 59-900 Zgorzelec, Poland; mwolski@mwolski.com.pl

**Keywords:** SPION, radio-labeled nanoparticles, anticancer therapy, magnetic hyperthermia, drug delivery, trastuzumab, superparamagnetic nanoparticles, multimodal therapy

## Abstract

Superparamagnetic iron oxide nanoparticles (SPIONs) are widely used for biomedical applications for their outstanding properties such as facile functionalization and doping with different metals, high surface-to-volume ratio, superparamagnetism, and biocompatibility. This study was designed to synthesize and investigate multifunctional nanoparticle conjugate to act as both a magnetic agent, anticancer immunological drug, and radiopharmaceutic for anticancer therapy. The carrier, ^166^Ho doped iron oxide, was coated with an Au layer, creating core-shell nanoparticles ([^166^Ho] Fe_3_O_4_@Au. These nanoparticles were subsequently modified with monoclonal antibody trastuzumab (Tmab) to target HER2+ receptors. We describe the radiobioconjugate preparation involving doping of a radioactive agent and attachment of the organic linker and drug to the SPIONs’ surface. The size of the SPIONs coated with an Au shell measured by transmission electron microscopy was about 15 nm. The bioconjugation of trastuzumab onto SPIONs was confirmed by thermogravimetric analysis, and the amount of two molecules per one nanoparticle was estimated with the use of radioiodinated [^131^I]Tmab. The synthesized bioconjugates showed that they are efficient heat mediators and also exhibit a cytotoxic effect toward SKOV-3 ovarian cancer cells expressing HER2 receptors. Prepared radiobioconjugates reveal the high potential for in vivo application of the proposed multimodal hybrid system, combined with magnetic hyperthermia and immunotherapy against cancer tissues.

## 1. Introduction

Superparamagnetic iron oxide nanoparticles (SPIONs) exhibit unique physicochemical properties [1,2,3], making them an attractive material for various biomedical applications including drug delivery [4,5,6], chemo-photothermal therapy [7,8], magnetic hyperthermia [9,10], magnetic resonance imaging (MRI) [11,12], and gene magnetofection [13,14]. Due to their properties, iron oxide-based nanoparticles, also known as ferumoxytol, have already gained approval for use as MRI contrast agents and as iron deficiency therapeutics by the Food and Drug Administration (FDA). Many different iron oxide-based nanoparticles have been evaluated over the years, for a wide variety of medical applications such as Feraheme^®^ [15], Feridex I.V.^®^ [16], and GastroMARK^®^ [17], and much more [10]. Thanks to their heat generation capability under the alternating magnetic field (AMF) they can work as efficient therapeutic agents.

The basis of hyperthermia relies on the fact that elevated temperatures localized within a tumor can damage and/or kill malignant cancer cells within the body. Numerous cell studies have shown that hyperthermia causes programmed cell death (apoptosis) as well as cell death (necrosis), depending on the type of the cell and on the temperature applied. Although the positive effect of heat in tumor treatment has been known for a long time, hyperthermia has not evolved noticeably due to the lack of appropriate heating methods able to localize the temperature increase solely in tumor tissue. Recently, this problem has been solved by the use of coated magnetic nanoparticles as a local heat sources [18,19,20]. This method, known as magnetic hyperthermia (MH), allows for the precise destruction of tumors by temperature increase, localized exclusively within the tumor tissue [21,22]. This is achieved by the accumulation of magnetic nanoparticles within such tissue and then, under the exposure of an alternating magnetic field (AMF), to make the nanoparticles localized sources of heat, there results the bulk heating of tumor tissue that leads to the targeted cell death [23,24]. However, as of late 2020, the only clinically approved magnetic hyperthermia (MH) therapy employs spherical aminosilane-coated iron oxide nanoparticles (NanoTherm, MagForce Nanotechnologies) to treat glioblastoma multiforme in patients and is currently in a clinical trial for prostate cancer [25,26]

However, currently emerging applications also make use of localized heating on the scale of single nanoparticles by, e.g., actuation of selected biochemical pathways [27] or thermosensitive drug release [28]. By precisely tuning the driving conditions of the AMF (amplitude, frequency, and time), we can control the local temperature rise to a level sufficient to induce tumor destruction.

Moreover, mild hyperthermia (application of 41–46 °C) can be combined with other antitumor therapies such as chemotherapy, endoradiotherapy, or even photodynamic therapy, which in a synergistic effect leads to killing more cancer cells and as a result, it also provides more effective treatment [29]. It is also considered to be well suited even for endoscopy treatment [30,31].

The synergetic effect of external ionizing radiation and hyperthermia in killing cancer cells is well-known since the discovery of cancer cells that are resistant to radiation but sensitive to hyperthermic conditions [32]. Combining both therapeutic concepts, one expects increased efficiency of the radiation treatment by prior application of hyperthermia, including magnetic hyperthermia, which could possibly decrease the delivered radiation dose. It is thought that tumor tissue is more hypoxic, more acidic, and nutrient-deficient compared to normal tissues [33]. Because radiation resistance is often observed in the same tumor regions, the temperature rise increases the effectiveness of radiotherapy and allows a reduction of the radiation dose [34]. As observed, mild hyperthermia causes an increase of the intratumoral blood flow, and subsequent tumor re-oxygenation facilitates the generation of reactive oxygen species by ionizing radiation, leading to increased DNA damage in the irradiated volume [34]. Similar effects can also be expected in the case of a combination of magnetic hyperthermia with targeted radionuclide therapy. Targeted radionuclide therapy involves a radioactive drug called a radiopharmaceutical that targets cancer cells [35]. Radiopharmaceuticals typically consist of a radioactive atom combined with a cell-targeting molecule that seeks and destroys cancer cells [36]. A significant advantage of the combination of magnetic hyperthermia with radionuclide therapy is a better concentration of the toxic effect of radiation on the cancerous tissue than in the case of a combination with endoradiotherapy [37]. In previous works, SPIONs have been labeled with a series of β- and α particle emitters including, ^90^Y [38], ^223^Ra [39], and ^225^Ac [40].

There are two strategies for the synthesis of radioactive SPIONs: incorporation into the structure, or surface functionalization (including the construction of shell layers). It is known that the lanthanide cations can exchange Fe^3+^ cations in the magnetite structure. Although the doping of SPIONs with lanthanides such as Gd^3+^ or Ho^3+^ by replacing Fe^3+^ in the Fe_3_O_4_ core usually reduces the magnetic properties, it makes it possible to label SPIONs with radionuclides such as ^166^Ho, ^161^Tb, or ^177^Lu. Our previous studies on the effect of Ho^3+^ doping on the magnetic properties of SPIONs have shown that the optimal Ho^3+^ doping is from 1 to 2.5 atomic % in relation to the total iron content [3]. Our further studies with a bioconjugate containing Ho^3+^ doped SPION combined with trastuzumab have shown high stability during alternating magnetic field application, which is a very important feature for their possible medical applications [41]. Holmium radioisotope ^166^Ho (*t*_1/2_ = 26.8 h) is attractive as it emits high-energy beta radiation, which can be used for a therapeutic effect and gamma radiation, which can be used for nuclear imaging purposes. Since the holmium element is monoisotopic (^165^Ho has a natural abundance of 100%), the target is relatively cheap and the only product in neutron irradiation is ^166^Ho. Various ^166^Ho radiopharmaceuticals are now used in cancer therapy, such as ^166^Ho-labelled microspheres for brachytherapy, ^166^Ho labeled chitosan for hepatocellular carcinoma, and ^166^Ho bisphosphonates for bone metastases [42,43].

Unfortunately, our preliminary studies with ^166^Ho labeled SPIONs showed a large 40% leakage of ^166^Ho into the solution, making it impossible for therapeutical applications. It is caused by the larger size of the ionic radius of Ho^3+^ in comparison to the Fe^3+^ cations. To prevent the leakage of ^166^Ho, we proposed to cover the ^166^Ho-labeled SPIONs with a thin layer of Au atoms creating ^166^Ho-SPION@Au core-shell nanoparticles.

In this work, we present a facile synthesis of radioactive SPIONs doped with ^166^Ho radioisotope and coated with a gold layer against holmium leaking from the structure of the magnetic core. To target cancer cells, ^166^Ho-SPION@Au nanoparticles were conjugated with a trastuzumab-monoclonal antibody, HER2 receptor antagonist. Obtained radiobioconjugate was tested for stability, dispersity, and receptor affinity. Then, the radiobioconjugate was tested with the use of magnetic hyperthermia (MH) to evaluate the specific rate absorption and optimize the MH parameters such as alternating magnetic field amplitude and frequency to reach the temperature required for the apoptosis of cells. Finally, cytotoxicity and apoptosis tests were performed to test the antitumor activity of the radiobioconjugate on cancer cells through the combined effects of β-radiation and magnetic hyperthermia.

## 2. Materials and Methods

### 2.1. Materials

Iron (III) chloride hexahydrate FeCl_3_·6H_2_O Aldrich ACS reagent 97%, and iron (II) chloride tetrahydrate FeCl_2_·4H_2_O puriss p.a. ≥99% (RT) were supplied from Sigma-Aldrich (St. Louis, MO, USA). Carrier added (c.a.) holmium-166 was obtained by neutron irradiation of solid HoCl_3_·6H_2_O at reactor Maria, National Center for Nuclear Research, Otwock-Świerk, Poland. Deionized water with resistivity of 18.2 MΩ·cm at 25 °C was obtained using the Milli-Q ultra-pure water filtering system from Merck. Synthesized SPIONs were modified with sodium tricitrate and gold with the use of chloroauric acid HAuCl_4_. Both compounds were purchased from Sigma-Aldrich (≥99.9% purity) (St. Louis, MO, USA). NaOH was supplied from POCH (POCH, Wrocław, Poland). HS-PEG-NHS ester 5k used for drug binding was purchased from NANOCS (PEGLAB, Boston, MA, USA). Trastuzumab (Herceptin) was supplied from Roche (Roche Pharma AG, Grenzach-Wyhlen, Germany). Sterile PBS buffer solution for biological use was purchased from VWR Life Science (Randor, PA, USA). MTS was supplied from Promega (Promega, Madison, WI, USA).

SKOV-3 (HER2-positive) and MDA-MB-231 (HER2-negative) cell lines were obtained from the American Type Culture Collection (ATCC, Rockville, MD, USA) and cultured in McCoy’s 5A and DMEM mediums, respectively. The cell culture mediums were enriched with 10% heat-inactivated fetal bovine serum, streptomycin (100 μg/mL), and penicillin (100 IU/mL). Cells were cultured in a humidified atmosphere with 5% CO_2_ at 37 °C according to ATCC protocol. Before their in vitro use, cells were detached using trypsin-EDTA (0.25%). All reagents for cell growth were purchased from Biological Industries (Beth Haemek, Israel).

### 2.2. Synthesis and Modification of [^166^Ho]Fe_3_O_4_

**Figure 1:**
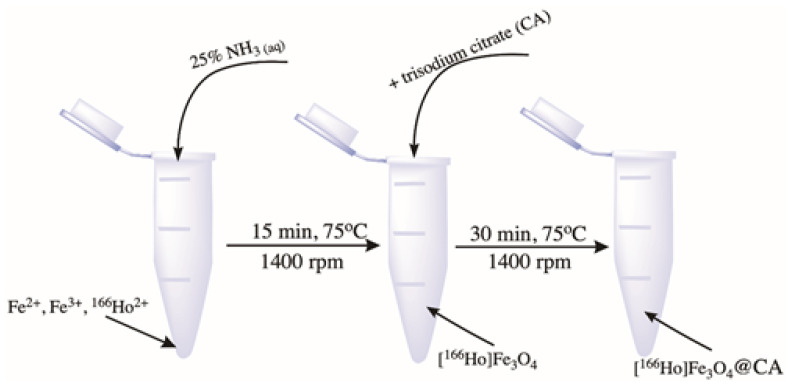
Scheme of precipitation synthesis and coating of [^166^Ho]Fe_3_O_4_.

The synthesis of radio-labeled SPIONs was performed using a co-precipitation technique based on our previous work in which non-radioactive holmium was incorporated into the superparamagnetic structure [41]. The [^166^Ho]Fe_3_O_4_ nanoparticles were synthesized from the following solutions: 53.249 mg FeCl_3_·6H_2_O in 500 µL of DI water, 19.881 mg of FeCl_2_·4H_2_O 250 µL of deionized (DI) water, and 1.192 mg of [^166^Ho]HoCl_3_·6H_2_O in 200 µL of DI water. Nanoparticles were synthesized by co-precipitation of these solutions with 28% NH_3(aq)_ solution in 2 mL Eppendorf type tube with a magnetic stirrer (1400 rpm) in 75 °C for 15 min, analogically to the synthesis performed for non-radioactive SPION. To stabilize the SPIONs, 110 mg of trisodium citrate dihydrate (CA) in 300 µL of DI water was added. The reaction was carried out for an additional 30 min with mixing and heating at 75 °C. The synthesis scheme is presented in Figure 1. The product was separated with magnetic sedimentation, washed two times with cold acetone, and dispersed in 1 mL of DI water.

### 2.3. Synthesis of Core-Shell [^166^Ho]Fe_3_O_4_@Au Nanoparticles

**Figure 2:**
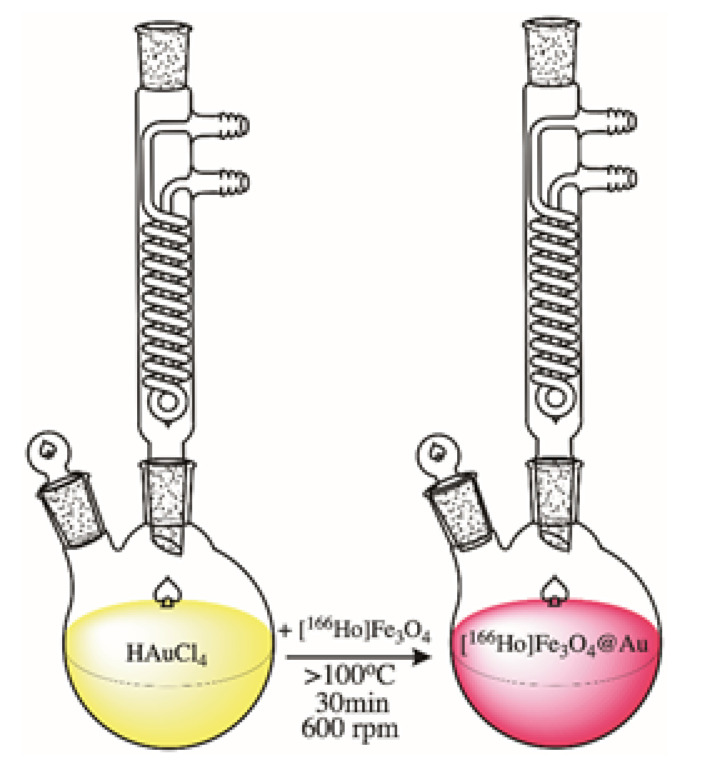
Schematic image of [^166^Ho]Fe_3_O_4_ coating with Au.

To synthesize the gold shell around citrate stabilized ^166^Ho doped Fe_3_O_4_ core, a modified method published by H. Zhou et al. was used [44]. Briefly, to a round bottom flask, 1.6-32 mg of AuCl_3_·3H_2_O solution in DI water was added with the pH set to 6–7 with 0.1 M NaOH. Gold chloride solution was set to boil under reflux with mixing at 600 rpm. When the boiling point was reached, the nanoparticles suspension was added. The coating reaction was performed for 30 min with continuous stirring. The product was purified by magnetic sedimentation, washed several times with DI water, and finally suspended in 1 mL. Figure 2 shows a schematic image of the Au-coating procedure.

### 2.4. Synthesis of [^166^Ho]Fe_3_O_4_@Au-Tmab Bioconjugate

**Figure 3:**
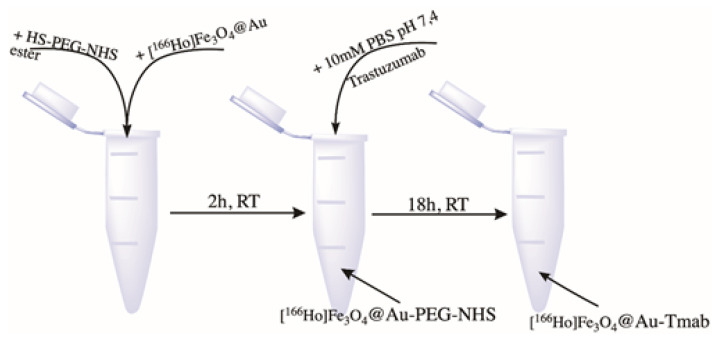
Scheme of [^166^Ho]Fe_3_O_4_ coating with Au.

In a 2 mL Eppendorf type tube, 1 mL of core-shell nanoparticles (NPs) was mixed with 5 mg of HS-PEG-NHS ester, 5 kDa. The reaction pH was set to 7 with 0.1 M NaOH. The pegylation reaction of the NPs was carried on a magnetic stirrer for 2 h at room temperature. The product was then separated from the unbound PEG linker by magnetic separation. After that, 500 µL of 10 mM PBS solution and 250 µg of trastuzumab were added to PEG-covered core-shell nanoparticles. A conjugation reaction was performed overnight at 1000 rpm and room temperature. Finally, bioconjugate was separated by magnetic sedimentation and washed several times with DI water. The product was stored at 4 °C in a lead casing for further studies. The scheme for radiobioconjugate synthesis is presented in Figure 3.

### 2.5. Quantification of the Number of Trastuzumab Molecules Per One Nanoparticle

The average number of Tmab molecules conjugated to each [^166^Ho]Fe_3_O_4_@Au nanoparticle was determined by a method based on coupling [^131^I]-labeled trastuzumab to nanoparticles by using the procedure described earlier by Cędrowska et al. [40] Briefly, 1 mg of trastuzumab in 200 µL of 0.01 M PBS was radioiodinated with ^131^I (10–15 MBq) by using tubes coated with 10 µg of dried Iodogen. After 10 min of incubation, the radioiodinated [^131^I]-trastuzumab was purified on PD-10 columns filled with Sephadex G-25 resin (GE Healthcare Life Sciences, Piscataway, NJ, USA). In the next step, 250 μg of [^131^I]-trastuzumab was added to cold [^166^Ho]Fe_3_O_4_@Au coated by HS-PEG-NHS ester-activated groups, and stirred overnight as described earlier. The next day, nanoparticles conjugated with [^131^I]-trastuzumab were separated from the solution using a solid magnet, washed several times, and resuspended in distilled water. The binding efficiency of [^131^I]-trastuzumab to [^166^Ho]Fe_3_O_4_@Au nanoparticles was assessed by measuring the proportion of radioactivity coupled to [^166^Ho]Fe_3_O_4_@Au to the total radioactivity added. Finally, to calculate the number of [^131^I]-trastuzumab attached to each [^166^Ho]Fe_3_O_4_@Au nanoparticle, the moles of the attached [^131^I]-trastuzumab were divided by the moles of the used nanoparticles.

### 2.6. Radiostability Studies

To perform the radiostability studies, 10 µL of freshly synthesized [^166^Ho]Fe_3_O_4_@Au (AuCl_3_·3H_2_O coating from 1.6–32 mg) nanoparticles and [^166^Ho]Fe_3_O_4_@Au-Tmab radiobioconjugate were added to 500 µL of DI water, human serum, and 0.9% NaCl solution to examine radio stability. Samples were incubated from 1 to 72 h in RT for saline and aqueous solutions and at 37 °C for human serum. For each measurement, 5 µL of the sample was mixed with 10 mM diethylenetriaminepentaacetic acid (DTPA) solution at 1:1 ratio (*v/v*) and placed onto iTLC plate. Additionally, 0.1 M citrate buffer pH 5.5 was used as a mobile phase (nanoparticles R_f_ = 0–0.1, free holmium-166 R_f_ = 0.8–0.9).

### 2.7. In Vitro Cytotoxicity Studies

Cytotoxicity studies were performed by MTS colorimetric assay for increasing the concentration of [Ho]Fe_3_O_4_@Au, [Ho]Fe_3_O_4_@Au-PEG, [Ho]Fe_3_O_4_@Au-Tmab (0.78–400 µg/mL) and c.a. ^166^HoCl_3_∙(0.05–30 MBq/mL). SKOV3 cells were seeded 24 h before the experiment in 96-well plates at a density of 2.0 × 10^3^ per well. Then the cells were washed with PBS and treated with increasing concentrations of the studied compounds, to a volume of 100 µL. Seeded cells were incubated with compounds for 18 h, washed twice with 100 µL of PBS, and incubated for another 24, 48, and 72h at 37 °C. Next, an MTS assay was added to each well, and plates were incubated for an additional 2 h at 37 °C in the dark. Lastly, the absorbance was measured at 490 nm using the Apollo 11LB913 microplate reader, Berthold (Bad Wildbad, Germany). The results are presented as cell viability (%) in comparison to the control not treated with the studied compounds.

### 2.8. Binding Specificity Studies

The binding affinity of [Ho]Fe_3_O_4_@Au-[^131^I]Tmab radiobioconjugates towards HER2 receptors was studied on SKOV-3 (HER2+) and MDA-MB-231 (HER2−) cell lines. Tested radiobioconjugate was incubated with cells in the presence (blocked) or absence (non-blocked) of 100× molar excess of trastuzumab. Briefly, 8 × 10^4^ cells per well were seeded onto 24-well plates and incubated for 24 h at 37 °C. After that, the cells were washed twice with PBS and radiobioconjugate solution was added to each well and incubated for 2 h at 4 °C. Next, the compound containing medium was removed and collected, cells were washed twice with cold PBS which was also collected. Finally, the cells were lysed with 1 M NaOH and the lysed fraction was collected as well. Unbound and bound fractions were measured on the Wizard 2^®^ automatic gamma counter.

### 2.9. Techniques

The morphology of SPIONs was determined with Transmission Electron Microscopy (TEM), Zeiss Libra 120 Plus, Stuttgart, Germany, operating at 120 kV. Complementary to TEM studies, the dynamic light scattering (DLS) method was used to analyze the hydrodynamic size of as-synthesized SPIONs and bioconjugated composite. DLS measurements were carried out with Malvern Instruments Zetasizer Nano ZS, Malvern, UK. The confirmation of successful bioconjugation was investigated using thermogravimetric analysis-(TGA Q50 (TA Instruments)), New Castle, PA, USA, under a nitrogen atmosphere. The magnetic hyperthermia (MH) experiments were performed with nanoScale Biomagnetics D5 Series equipment with CAL1 CoilSet. The specific absorption rate (SAR) values were estimated using MaNIaC Controller software (nB nanoScale Biomagnetics, Zaragoza, Spain).

## 3. Results and Discussion

### 3.1. Synthesis and Structural Characterization

The ^166^Ho@Fe_3_O_4_ nanoparticles were successfully prepared by the co-precipitation method. We can expect that Ho^3+^ analogously incorporates into Fe_3_O_4_ structure as it was observed previously in the synthesis of SPIONs doped with non-radioactive Ho^3+^ cations [9]. Based on X-ray studies, authors found that up to 2.5% of Ho^3+^ content, the crystalline structure of Fe_3_O_4_, was retained, in which some of the Fe^3+^ atoms were replaced by Ho^3+^. A schematic reaction taking place during the synthesis of doped SPIONs with ^166^Ho^3+^ should be as follows:

Fe^2+^ + (2 − x)Fe^3+^ + xHo^3+^ + 8OH^−^ →FeHo_x_Fe_2−x_O_4_ + 4H_2_O (1)

The efficiency of ^166^Ho incorporation into Fe_3_O_4_ samples was 91.62 ± 6.35%. To prevent leakage of ^166^Ho, the obtained ^166^HoFe_3_O_4_ samples were covered with a gold layer, and then the monoclonal antibody was attached via a PEG linker. The coverage efficiency of the gold attachment was 95–99% and the efficiency of trastuzumab binding was 49.57%.

**Figure 4:**
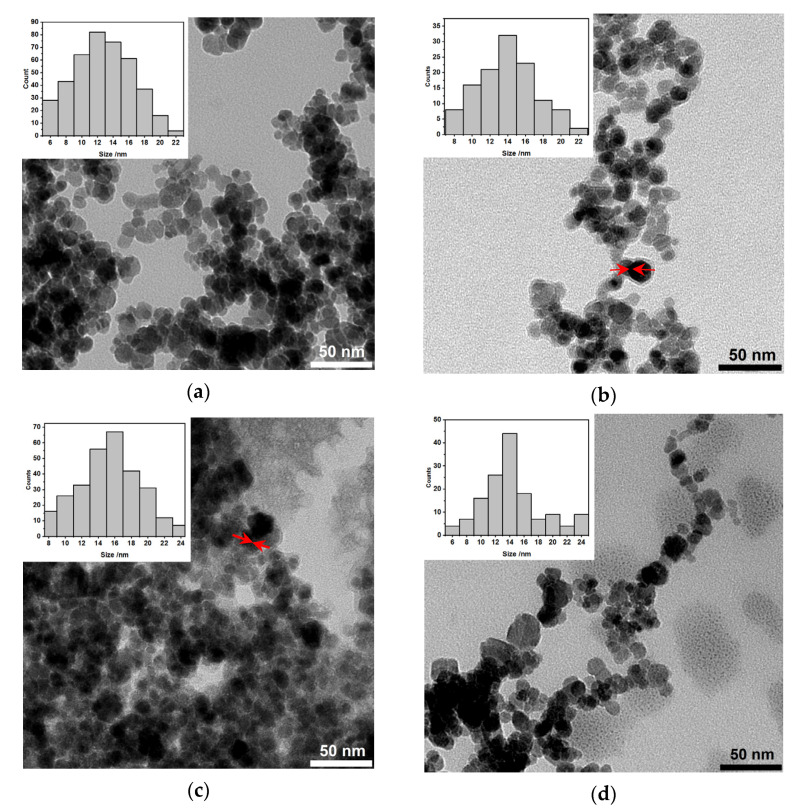
Transmission Electron Microscopy images of NPs: (**a**) Citrate stabilized [Ho]Fe_3_O_4_@CA and [Ho]Fe_3_O_4_@Au; (**b**) [Ho]Fe_3_O_4_@Au coated with PEG linker, before the attachment of Tmab; (**c**) [Ho]Fe_3_O_4_@Au-Tmab radiobioconjugates (PEG linker presence is not mentioned for clarity); and (**d**) [Ho]Fe_3_O_4_@Au-Tmab bioconjugates after magnetic hyperthermia treatment. Red arrows (**b**,**c**) point to the PEG or PEG-Tmab “misty” corona surrounding the [Ho]Fe_3_O_4_@Au core.

The morphology studies of synthesized structures were performed with the transmission electron microscopy (TEM). As can be seen in Figure 4a, there are core-based holmium doped SPIONs coated with gold (darker objects) and some that remained uncovered with Au (grey structures, stabilized with citrates only). The latter can be caused by the gold agglomeration around the SPIONs in aqueous media. They have well-defined and roughly spherical shapes with a measured average diameter below 20 nm. The gold covers the nanoparticles on the whole surface. Figure 4b presents the SPIONs that were additionally coated with PEG. Despite the presence of some bare SPIONs, the sample contains many nanoparticles that are coated with gold. The organic (PEG) shell is visible as a somehow “misty” ring surrounding each nanoparticle (red arrows). In the case of additional bioconjugation of PEG-coated SPIONs, the trastuzumab is present as the light greyish layer around the whole surface, coating all nanoparticles, see Figure 4c, red arrows. The following image presents the bioconjugate after the magnetic hyperthermia studies, see Figure 4d. As can be seen in this image, the drug is released from the surface.

Further studies show the hydrodynamic size of the nanoparticles measured by the dynamic light scattering technique are complementary to the TEM analysis. Measurements were performed in 1mM phosphate buffer saline pH 7.5 (PBS). As can be seen in Table 1 for measurements, the size of bare NPs evaluated by DLS was notably larger than that measured by TEM (10.3 ± 1.2 nm). The difference was observed because TEM and DLS are different techniques, since DLS determines the hydrodynamic diameter which includes the solvation layers. In the case of the TEM technique dehydration of the nanoparticles’ surface takes place in the vacuum environment of TEM and the diameter of bare nanoparticles is measured. The following results indicate the same tendency confirming hydrophilic properties of the conjugate. Au-coated NPs are about 50% larger than bare SPIONs, suggesting that gold forms a shell rather than biding to SPIONs in the form of nanoparticles. Next, the hydrodynamic size of the bioconjugate is above 90 nm confirming the presence of larger objects onto the SPIONs’ surface. Additionally, the zeta potential was measured to estimate the presence of the functional groups on the SPIONs’ surface. Bare SPIONs have a negatively charged oxide surface. When SPIONs are coated with gold their surface potential is shifted towards highly negative values (−42.6 ± 6.1 mV). The reduction of gold and oxidation of surface-bound citrate still gives a negative surface potential (−43.8 ± 6.95 mV). Attachment of HS-PEG-NHS ester shifts zeta potential towards more positive values (−39.5 ± 5.6 mV), however within the standard deviation. After conjugation of 149 kDa positively charged protein, the surface potential shifts significantly to −25.2 ± 5.21 mV. High standard deviation values are probably caused by a high concentration of NPs, ionic strength of solvent, and possible aggregation. However, surface potential values lower than −30.0 mV indicate that obtained NPs are stable in the studied medium.

### 3.2. UV-Vis Spectrometry Studies

**Figure 5:**
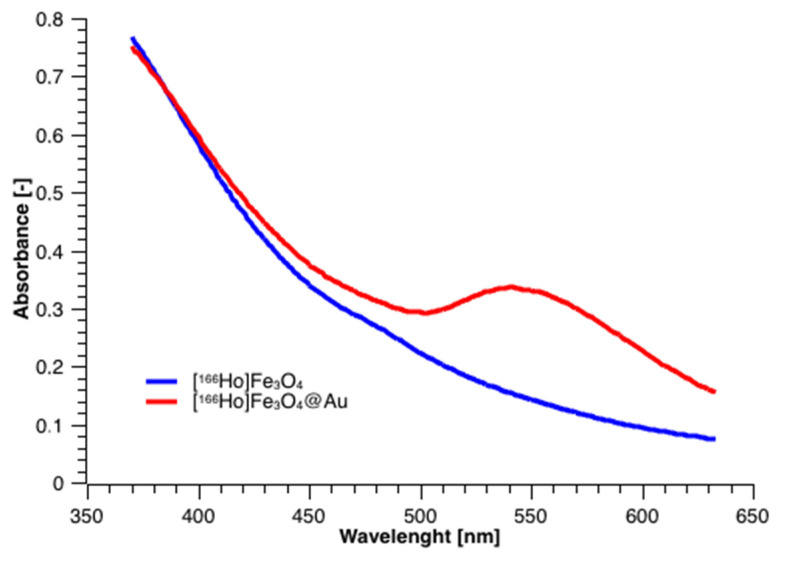
UV-vis spectra for [^166^Ho]Fe_3_O_4_ and [^166^Ho]Fe_3_O_4_@Au.

UV-Vis spectra were acquired for nanoparticles before and after coating to check for the presence of Au plasmon absorption with a characteristic peak. The results presented in Figure 5 show two spectra, where the blue curve corresponds to the bare SPIONs, and the red corresponds to the SPIONs coated with gold, respectively. The visible broad band, having a maximum absorption at about 550 nm, is characteristic of the gold presence on the surface. The presence of that band proves the formation of a thin film of metallic gold around [^166^Ho]Fe_3_O_4_ core. Depending on the film thickness and particle size, the color of the NPs’ suspension can vary from brown through red, and purple to blue [45,46], depending on the NP size.

### 3.3. Thermogravimetric Analysis

**Figure 6:**
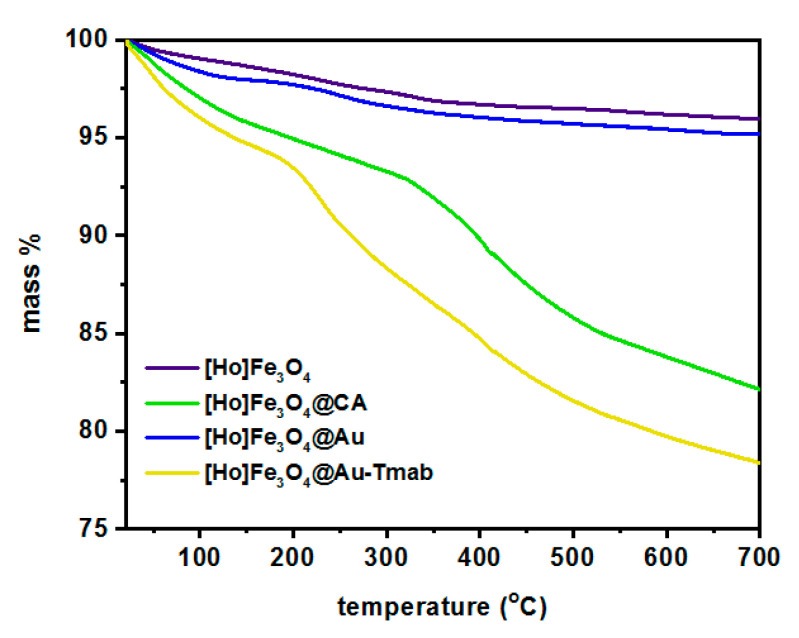
Thermograms of [Ho]Fe_3_O_4_, [Ho]Fe_3_O_4_@CA, [Ho]Fe_3_O_4_@Au, and [Ho]Fe_3_O_4_@Au-Tmab.

The attachment of PEG-NHS and Tmab to [^166^Ho]Fe_3_O_4_@Au was confirmed with thermogravimetric analysis (TGA) under the ambient atmosphere in the temperature range from room temperature to 650 °C, with a heating rate of 10 °C/min. The gradual mass loss while heating can be related to the decomposition of organic compounds modifying the surface of the nanoparticles. As can be seen in Figure 6, the mass loss for bare SPIONs is ca. 3% and can be caused by the presence of the water adsorbed in the sample. Gradual further mass loss of [^166^Ho]Fe_3_O_4_@CA is about 18% due to the presence of organic citrates on the SPIONs’ surface. In the case of gold-covered SPIONs ([Ho]Fe_3_O_4_@Au), the mass decrease with heating is about 4%, closer to the bare SPIONs. It is caused by the fact that a substantial amount of citrates were used for Au^3+^ reduction on the iron oxide surface. In the case of the [Ho]Fe_3_O_4_@Au-Tmab sample, we see a large decrease in mass due to decomposition of the attached protein and linker agent indicating the successful attachment of PEG linker and Tmab to the nanoparticle.

Since the linker and Tmab decompose together, the thermogravimetry technique was not suitable for the estimation of the amount of antibody on the surface of the nanocarrier. Therefore, we used ^131^I-labeled trastuzumab during the synthesis of bioconjugates.

### 3.4. Estimation of the Number of Attached Trastuzumab Per One Magnetite Nanoparticle

To determine the number of Tmab molecules attached to one nanoparticle, [Ho]Fe_3_O_4_@Au synthesis of bioconjugate using ^131^I-labeled trastuzumab was performed. Based on the obtained results, the amount of [^131^I]Tmab per mg of Fe_3_O_4_ core was found to be 118.93 μg with a coupling efficiency of 49.57 ± 6.23%. Having this value, the number of bound trastuzumab molecules per one [Ho]Fe_3_O_4_@Au nanoparticle was calculated. The calculations were carried out assuming that each nanoparticle is spherical and has a diameter of 18 nm (measured by TEM) and the magnetite density is 5.2 g/cm^3^ (we neglected the mass of attached Au). The obtained results indicate that approximately 2 trastuzumab molecules were coupled to one [Ho]Fe_3_O_4_@Au nanoparticle.

### 3.5. Hyperthermia Studies

Magnetic properties of the SPIONs doped with holmium conjugated with trastuzumab were studied within our previous work [41], giving a foundation for further exploration of bioconjugates based on [Ho]Fe_3_O_4_@Au NPs in the magnetic hyperthermia. In this work, magnetic hyperthermia studies were performed for [^166^Ho]Fe_3_O_4_@Au-Tmab radiobioconjugate in the human serum. The medium was chosen to have similar viscosity to the biological fluids, so the 0.5 mL of the aqueous suspension, having a density of 11 mg/mL, was inserted into the thermostated copper coil and the temperature changes were measured with an alternating magnetic field in the frequency range from 345.45 kHz to 759.05 kHz and with an amplitude from 75 G up to 200 G. Measurements were performed to reach the 45 °C (318 K) with a measuring range of error ±0.5 °C, and the SAR value along with the heating rate of the suspension were measured. The most common parameter allowing estimation of heat conversion efficacy of the sample is SAR, defined as the power P [W] generated per mass m [g] of nanoparticles. Such parameters depend on the magnetic field H [kA/m] and frequency of the magnetic field f [Hz], although the intrinsic loss power ILP parameter is also widely used: ILP = SAR/f∗H^2^ [47].

**Figure 7:**
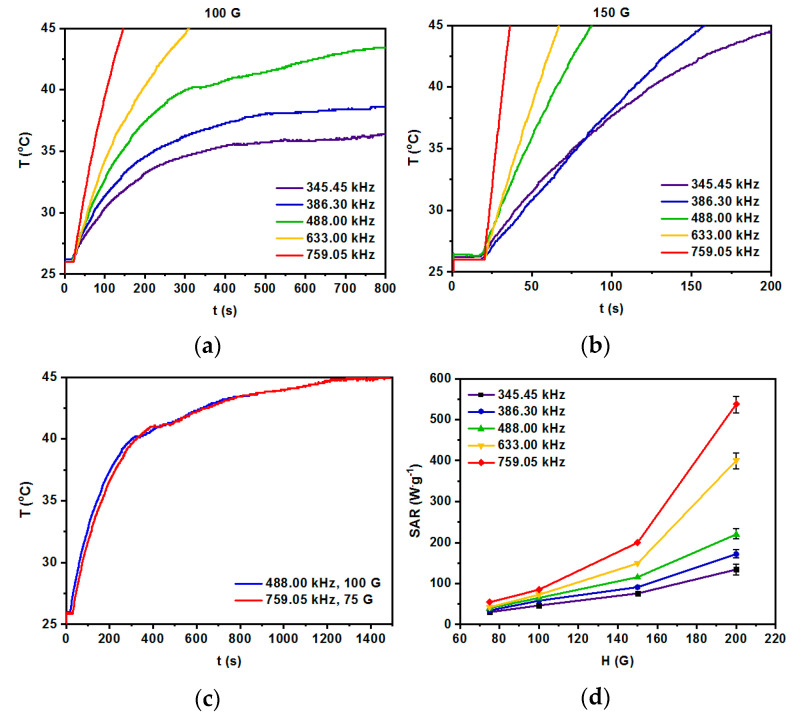
Heating of radio-labeled [Ho]Fe_3_O_4_@Au-Tmab bioconjugate in various ranges of frequency of magnetic field: (**a**) 100 G, (**b**) 150 G, (**c**) 75–150 G, and (**d**) dependence of SAR for various frequencies of the magnetic field as a function of the amplitude of the magnetic field. S.D. bar represents the largest value of five independent experiments.

As can be seen in Figure 7a,b, the temperature of the suspension for 100 G and 150 G at various frequencies of alternating magnetic field increases as a function of time. The fastest rise was generated for the highest frequency of the magnetic field (759.05 kHz), while a decrease of such parameters leads to the decrease of the measured temperature. From a biomedical point of view, the sample should reach a plateau at the temperature in the range 42–46 °C and it should be kept constant for several minutes with a frequency of the alternating magnetic field and its amplitude as low as possible, due to the Brezovich limit for such therapy [48,49]. Figure 7c shows optimal parameters for attaining the plateau at 45 °C are 759.05 kHz along with 75 G, and 488.00 kHz with 100 G for which the SAR values are about 54.3 and 72.7 W/g, respectively. The SAR values shown in Figure 7d were calculated using a ZaR subprogram of MaNIaC 1.0 Software, Nanoscale Biomagnetics, Zaragoza, Spain, with the following equation:

(2) $$\mathrm{SAR}=\frac{d_{l}\cdot {\mathrm{Ce}}_{l}}{N_{p}}{\left(\frac{\mathrm{dT}}{\mathrm{dt}}\right)}_{\max}$$

d_l_—dispersant density [kg/m^3^]; Ce_l_—dispersant specific heat [kcal/kg °C]; N_p_—nanoparticles density [kg/m^3^]; T—temperature [^o^C], and t—time [s].

The majority of current literature reports pristine nanoparticles of the different core compositions. However, there are also numerous reports on surface-modified SPIONs [50]. Several of these cited literatures even report on magnetic nanoparticles covered with phosphorylated mPEG or thiolated PEG. The reported SAR values are within the range of 85 W/g (hydroxyapatite-coated SPIONs [51]), through 248 W/g (for thiolated PEG terminated with folic acid as targeting vector molecule [52]), to as high as 3050 W/g [53], or 1018 W/g for Mn_0.6_Zn_0.4_Fe_2_O_4_ nanospheres [54]. All the reported SAR values, apart from the field intensities and frequencies used in these works, depended strongly on size, shape, and the content of the core.

However, to the best of our knowledge, there were no further constructs, enlarging the thickness of the shell covering the iron oxide nanoparticles. Therefore, it is difficult to compare the SAR values of our new bioconjugates with the reported data. Nevertheless, we are inclined to consider our results quite reasonable, referring them to the work on nanoparticle nanoclusters of 50–100 nm, coated with thiolated PEG with folic acid (FA) vector [52]. The value of SAR = 248 W/g reported in their work, was obtained for the product of field intensity × frequency equal to 1.8 × 10^9^ A·m^−1^·s^−1^ being below the Brezovich physiological limit (5 × 10^9^ A·m^−1^·s^−1^). Our value of SAR = 72.3 W/g obtained for nanoparticles each covered separately with a PEG–Tmab organic shell, is ca. three times smaller (for 3.9 × 10^9^ A·m^−1^·s^−1^). Nevertheless, we feel quite confident that the synthesized radiobioconjugates are efficient heat mediators for the further studies described below.

### 3.6. Stability of Radiobioconjugates

**Figure 8:**
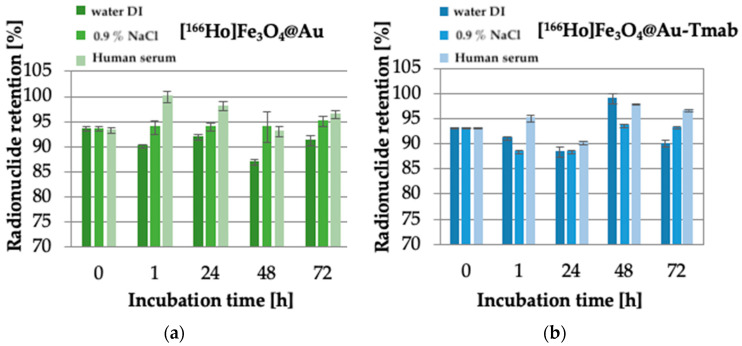
Stability studies of: (**a**) [^166^Ho]Fe_3_O_4_@Au, (**b**) [^166^Ho]Fe_3_O_4_@Au-Tmab in water DI, 0.9% NaCl and human serum. Data are expressed as mean ± SD (*n* = 3).

Radiobioconjugate [^166^Ho]Fe_3_O_4_@Tmab was characterized by low stability due to the radioisotope leakage from the core of the nanoparticles as high as 40% after 24 h of incubation in 0.9% NaCl. Due to that, we proposed synthesizing a protective gold shell to prevent said leakage. The obtained results of the stability of a gold shell-protected core and radiobioconjugate are presented in Figure 8.

Obtained stability results are comparable for both tested compounds-core-shell nanoparticle and radiobioconjugate, as well as for all used solutions. No significant changes in core stability were observed at examined time intervals, and the maximum observed leakage did not exceed ~13%. This leads to the conclusion that the proposed protective gold shell provides stabilization of the core doped with radioactive holmium and prevents leakage up to 72 h of incubation in various media at the studied radioactivities. Additionally, the coating provides an opportunity for easy modification of the surface of nanoparticles with a thiol-reactive agent with a known affinity towards gold surfaces. Radiobioconjugate is characterized by overall high stability (>90–95%) in human serum, which is crucial for further application as a radiopharmaceutical. However, the gold-coating procedure carried out under rather harsh conditions (see p. 2.3) resulted in a decrease of specific activity of the obtained radionuclide-labeled nanoparticles to the level of up to 30 MBq/mL. Unfortunately, such a low specific activity makes it impossible to carry out biological tests, even on cells.

Therefore, in further studies, we limited the cytotoxicity tests only to the non-radioactive bioconjugates. In this case, we expected the cytotoxicity to be related to magnetic hyperthermia and immunological properties of Tmab.

### 3.7. In Vitro Cytotoxicity Results

**Figure 9:**
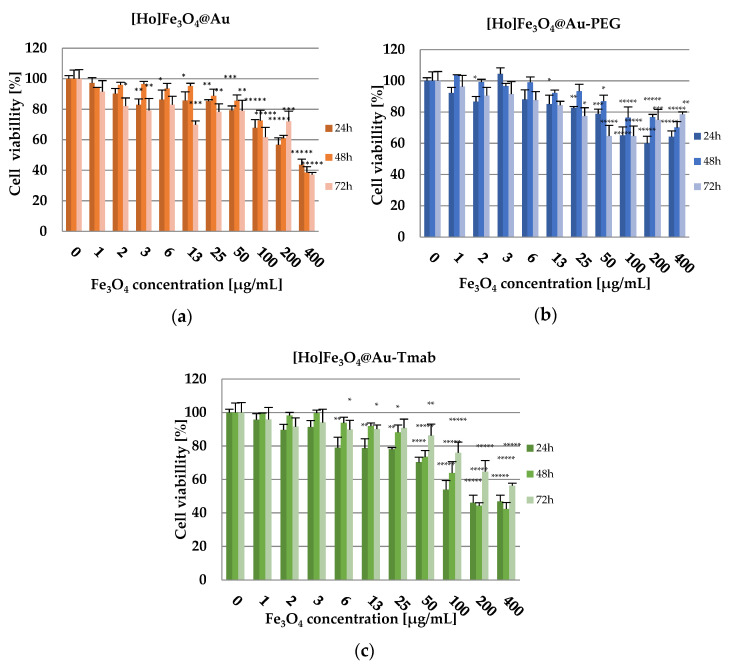
Cell viability after treatment with different concentrations of: (**a**) [Ho]Fe_3_O_4_@Au, (**b**) [Ho]Fe_3_O_4_@Au-PEG, and (**c**) [Ho]Fe_3_O_4_@Au-Tmab. SKOV-3 were incubated for 24, 48, and 72 h, after which their viability was determined by MTS assay. The results are expressed as a percentage of control cells. Data are expressed as the mean ± SD (*n* = 3). Statistical significance was considered if *p* < 0.05 (*), *p* < 0.01 (**), *p* < 0.001 (***), *p* < 0.0001 (****), *p* < 0.00001 (*****).

The results of cytotoxicity obtained via MTS colorimetric assay are presented in Figure 9a–c. Cytotoxicity is displayed as cell viability percentage of cells, treated with nanoparticles suspension in comparison to non-treated cells incubated with medium only. The viability of the control group is set to 100%. Tests were performed on SKOV-3 (HER2+).

The obtained results of cytotoxicity studies (Figure 9) indicate that [Ho]Fe_3_O_4_ nanoparticles exhibit a toxic effect in a dose-dependent manner starting with a Fe_3_O_4_ concentration at 100.00 µg/mL (cell viability at 75.91 ± 2.35% after 72 h of incubation), reaching only 37.03 ± 1.51% of cell viability after 72 h of incubation for the highest tested dose, 400.00 µg/mL). This effect may be caused by sedimentation of non-stabilized, agglomerating nanoparticles, long incubation time, and static conditions of the conducted experiment. However, stabilized [Ho]Fe_3_O_4_@Au-PEG nanoparticles have only a small toxic effect on the viability of the SKOV-3 cells (highest recorded toxicity with cell viability equal to 60.01 ± 2.72% after 24 h of incubation for the 200.00 µg/mL concentration of the compound), especially in comparison to bare [Ho]Fe_3_O_4_@Au. At the same time, [Ho]Fe_3_O_4_@Au-Tmab bioconjugate reduced the metabolic activity of SKOV-3 cells, in a dose-dependent manner, to 42.36 ± 2.58% at 48 h for the highest concentration of the tested compound. This result is probably caused by the cytotoxic effect of the conjugated monoclonal antibody-trastuzumab. Obtained results for tested Ho-[Ho]Fe_3_O_4_@Au-Tmab bioconjugate demonstrate higher toxicity than trastuzumab alone and also SPION nanoparticles without an @Au shell, which is presented in a previously published paper [41]. Thus, we can see that the Tmab in the bioconjugate plays not only a targeting role but also increases the cytotoxicity of nanoparticles.

### 3.8. Binding Affinity

**Figure 10:**
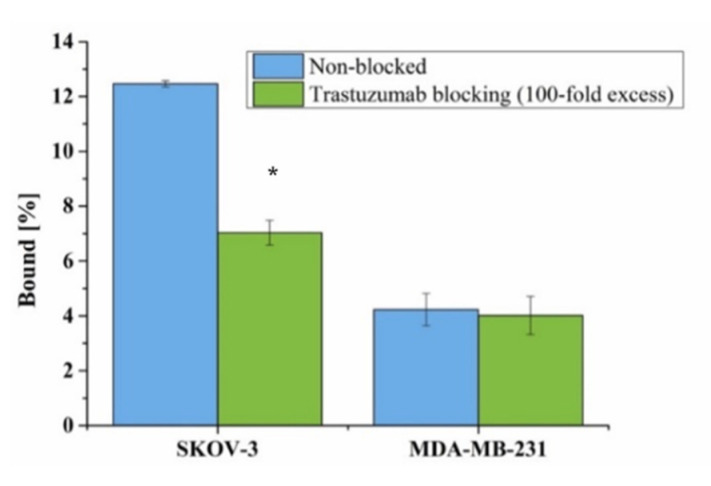
Specificity of binding on SKOV-3 (HER2+) and MDA-MB-231 (HER2−). [Ho]Fe_3_O_4_@Au-[^131^I]Tmab was incubated in the presence and absence of 100x molar fold of free trastuzumab. Data are expressed as a mean ± SD (*n* = 3). Statistical significance was considered if *p* < 0.05 (*).

The binding affinity of [Ho]Fe_3_O_4_-[^131^I]Tmab radiobioconjugate to HER2 receptors was examined on SKOV-3 (HER2+) and MDA-MB-231 (HER2−) cell lines. Tested radiobioconjugate was incubated in the presence (blocked) or absence of a 100-fold molar excess of non-labeled trastuzumab. The results are presented in Figure 10.

A significant difference (5.43%, *p* < 0.05) in the binding of radiobioconjugate was observed between non-blocked (12.47 ± 0.11%) and blocked (7.04 ± 0.45%) trastuzumab SKOV-3, which indicates specific binding (Figure 10). As expected, no such significant difference (*p* > 0.05) was observed for the HER2− blocked (4.23 ± 0.59%) and non-blocked (4.01 ± 0.70%) MDA-MB-231 cell line. This lack of binding for the used HER2− cell line additionally confirms the specificity of the binding of tested radiobioconjugate. Unfortunately, as expected, a high background coming from radiobioconjugate was observed for both tested cell lines similar to our previous experiences with trastuzumab modified barium ferrite NPs [55]. Binding of [Ho]Fe_3_O_4_@Au-[^131^I]Tmab to MDA-MB-231 and blocked SKOV-3 cells can be caused by sedimentation of nanoparticles in cell culture media due to opsonization and a long incubation period during the experiment, as the studies were performed in static conditions. However, specificity of binding was found similar to other trastuzumab-modified nanoparticles [55,56].

## 4. Conclusions

Superparamagnetic iron oxide nanoparticles doped with β- emitter ^166^Ho were synthesized, coated with gold nanoparticles, and conjugated with a monoclonal antibody as a potential agent for combined targeted radionuclide therapy, and magnetic hyperthermia. We showed that covering [^166^Ho]Fe_3_O_4_ nanoparticles with a layer of gold stabilizes ^166^Ho in the samples. However, the selected procedure results in a large decrease of specific activity of ^166^Ho in nanoparticles. We are now recognizing other, milder synthetic procedures resulting in higher radioactivity of ^166^Ho in the radiobioconjugate. The demonstrated high receptor affinity of the radiobioconjugate should allow the cumulation of nanoparticles in the targeted site. Trastuzumab-modified bioconjugates exhibited higher cytotoxicity than non-targeted [Ho]Fe_3_O_4_@Au-PEG conjugates. This effect against SKOV-3 cells indicates that [^166^Ho]Fe_3_O_4_@Au-Tmab radiobioconjugate, after the suitable redesign of synthesis to prevent radioactivity loss, can be a potent therapeutic agent against cancerous cells exhibiting overexpression of HER2 receptors, including breast and ovarian tumors. In addition, the confirmed superparamagnetic properties of radiobioconjugates allow for reaching temperatures used in mild hyperthermia and also make them suitable for guided delivery to the target tissues under the influence of an external magnetic field. The obtained results show that trastuzumab conjugated nanoparticles could be potentially used for the synergistic treatment of HER2+ breast and ovarian cancers. The designed NPs proved their binding specificity, also being comparable with other similar radiobioconjugates synthesized by our research group [55,56,57].

## Figures and Tables

**Table 1:** Values of hydrodynamic diameter and zeta potential of nanoparticles measured in PBS.

Sample	Hydrodynamic Diameter [nm]	Zeta Potential [mV]	Polydispersity Index
[Ho]Fe_3_O_4_@CA	36.1 ± 16.0	−42.6 ± 6.1	0.240
[Ho]Fe_3_O_4_@Au	64.8 ± 30.0	−43.8 ± 7.0	0.223
[Ho]Fe_3_O_4_@Au-PEG	55.5 ± 26.2	−39.5 ± 5.6	0.190
[Ho]Fe_3_O_4_@Au-Tmab	113.8 ± 28.8	−25.2 ± 5.2	0.378

## Data Availability

Not applicable.
